# The ontological status of behaviour: lessons from experimental neurosis (1900–1950)

**DOI:** 10.1007/s40656-026-00746-1

**Published:** 2026-07-08

**Authors:** Rodrigo J. De Marco

**Affiliations:** School of Biological and Environmental Sciences, Faculty of Health, Innovation, Technology and Science, Liverpool John Moores University, Byrom Street, Liverpool, L3 3AF United Kingdom

**Keywords:** Behaviour, Animal models, Behavioural dysregulation, Experimental neurosis, Psychopathology, Translational research

## Abstract

This article examines how early twentieth-century comparative psychology treated behaviour as a primary site for studying regulation, dysfunction, and adaptation, using experimental neurosis as a historical case study. Beginning with Pavlov’s observation that dogs exposed to irresolvable conflict developed persistent behavioural and autonomic disturbances, the paradigm expanded through mid-century work by Liddell, Gantt, and Masserman, who introduced new species, longitudinal designs, and ecologically grounded conditions revealing dysregulation as a multilevel, temporally extended phenomenon. In this tradition, behaviour was not construed as mechanistic output but as an autonomous level of organisation whose structured patterns, i.e., variability, context-sensitivity, stability, and breakdown, made internal regulatory dynamics experimentally accessible in ways physiology alone could not. Hybrid methodologies integrating laboratory control with naturalistic observation showed that pathological breakdown provides access to organisational features obscured during normal function. By mid-century, however, this behaviourally rich framework was displaced by reductionist approaches based on standardised assays and physiological endpoints. Recent historiography documents this shift as an epistemological transformation in which behaviour was redefined from interface to index, from organised system to dependent variable. These tensions persist in contemporary neuroscience and translational research, where behaviour is routinely subordinated to mechanism despite gaps between circuit-level manipulation and behavioural explanation. This historical analysis supports the claim that mechanistic approaches which bypass behaviour incur explanatory loss, eliminating the domain in which neurophysiological processes acquire functional meaning. Progress in understanding psychopathology requires restoring behaviour to ontological and epistemological centrality as the irreducible interface through which adaptive and pathological regulation become scientifically accessible.

## Introduction

Contemporary neuroscience faces a persistent conceptual difficulty. Behaviour is routinely treated as a downstream output of neurophysiological mechanisms rather than as the primary phenomenon through which those mechanisms become scientifically accessible (Krakauer et al., 2017; Bechtel, 2008; Jonas & Kording, 2017; Gomez-Marin & Ghazanfar, 2019). This tendency is uneven rather than universal. It is most pronounced in circuit-first and assay-based programmes—optogenetic or chemogenetic dissection oriented primarily to identifying mechanism, and high-throughput screening that treats standardised tests as proxies for psychiatric states—even as a growing body of work in computational ethology and naturalistic neuroscience, including several of the critiques cited above, presses in the opposite direction. Where it operates, however, it is not merely a methodological tendency, but an ontological stance. By subordinating behaviour to mechanism, contemporary frameworks risk eliminating the very phenomena that render internal regulatory dynamics intelligible. Behaviour is not a passive readout of deeper processes; it is the interface through which those processes manifest and can be known. Reducing behaviour to “mere output” constitutes a form of ontological damage (Lorenz, 1973), severing access to the regulatory organisation that scientific explanation requires.

This article develops that argument through a historical case study. In the early twentieth century, the emergence of animal models of psychopathology was part of a broader effort to access mental phenomena experimentally through behaviour. Comparative psychology and Pavlovian physiology—developing in parallel within a Darwinian framework of continuity across species (Boakes, 1984; Richards, 1987; Terrace, 1987)—treated behaviour as an experimentally tractable and conceptually rich domain capable of bridging mind, brain, and adaptive function. Scientists such as Conwy Lloyd Morgan (1852–1936), Edward L. Thorndike (1874–1949), and Ivan P. Pavlov (1849–1936) approached behaviour neither as a superficial display nor a dispensable proxy, but as the primary datum through which internal states and regulatory dynamics could be inferred.

Pavlov’s concept of the conditioned reflex (Pavlov, 1928) embodied this stance. Yet by the 1920s, he and his collaborators reported phenomena that exceeded simple stimulus-response models: dogs faced with irresolvable discrimination tasks or prolonged motivational conflict developed persistent disturbances—withdrawal, agitation, autonomic instability—that Pavlov termed “experimental neurosis” (Pavlov, 1941). These were not artefacts of inadequate control, but indicators that behaviour is dynamically regulated, and vulnerable to breakdown under chronic conflict and stress. Experimental neurosis marked a conceptual turning point. Behavioural dysregulation became a window onto the organisation—and disorganisation—of internal regulatory processes that could not be observed directly.

Extending this programme, researchers such as Howard S. Liddell (1895–1962), W. Horsley Gantt (1892–1980), and Jules Masserman (1905–1998) combined laboratory control with naturalistic observation, treating animals not as mechanistic devices but as organisms whose behavioural organisation, variability, and breakdown revealed the dynamics underlying adaptive function (Liddell et al., 1936; Anderson & Parmenter 1941; Masserman, 1943a; Gantt, 1944; Ramsden, 2018; Liddell, 1947). Their work made behaviour not simply a dependent measure, but the foundation for theorising regulation, conflict, emotion, and pathological breakdown.

By mid-century, however, this behaviourally rich framework began to give way to more reductionist paradigms, as emphasis shifted toward physiological markers of stress (Selye, 1950). Over the following decades this trajectory produced a battery of standardised behavioural assays in stress research and behavioural pharmacology (e.g., forced swim test: Porsolt et al., 1977; elevated plus maze: Pellow et al., 1985; see Cryan et al., 2002), privileging simplified, readily quantified outputs. This shift brought real advantages: standardised assays delivered reproducibility, cross-laboratory comparability, and the throughput on which pharmacological screening depended—advantages the labour-intensive, individualised methods they displaced could not match. The cost was conceptual rather than technical. Behaviour was increasingly treated as an index of internal states rather than the level at which regulatory dynamics could be studied in their own right (Garner, 2014). Historical accounts stress the depth of this conceptual shift. Kirk and Ramsden (2018) show that Liddell’s Cornell Behaviour Farm functioned as a hybrid experimental space in which physiological, psychological, and ethological methods were deliberately integrated, treating animals as individuals whose emotional lives demanded systematic attention. Koch (2019) traces how the meaning of experimental neurosis evolved from the 1920s to the 1970s, showing that the field’s move to standardised laboratory rats corresponded with a narrowing of theoretical focus, in which emotion, subjectivity, and behavioural meaning were displaced by neo-behaviourist framings. Ramsden (2011) similarly documents how crowding and stress research at the National Institute of Mental Health exemplified this transformation toward simplified behavioural endpoints and standardised conditions.

This historical trajectory provides a revealing lens through which to examine contemporary conceptual problems. The systematic subordination of behaviour to mechanism continues to shape animal models of psychopathology (Willner, 1984; Cryan et al., 2002; Pawlak et al., 2008), translational psychiatry (Insel, 2009, 2010; Insel & Cuthbert, 2015), and systems neuroscience more broadly. Yet behaviour remains the only domain through which internal regulatory organisation—its flexibility, variability, context-sensitivity, and breakdown—can be accessed. Behaviour is not what lies after mechanism; it is what renders mechanism explanatory in the first place.

Here, I use early comparative psychopathology, and experimental neurosis in particular, to reconstruct a moment in which behaviour held primary ontological and epistemological status. The paper first establishes the conceptual landscape of early comparative psychology, examines Pavlov’s experimental neurosis and its expansion, analyses how neurosis was reconceptualised, traces connections to clinical psychiatry, and draws epistemological lessons for contemporary neuroscience. The aim is not to advocate a return to past techniques, but to examine the conceptual possibilities available when behaviour was treated as the essential interface through which internal dynamics could be inferred. The historical case serves to illuminate a broader claim: certain forms of mechanistic reduction eliminate the very phenomena that scientific explanation depends upon, and reintegrating behaviour as a primary level of analysis is crucial for bridging physiological, neuroendocrine, and neural findings with models of adaptive and pathological regulation.

## From evolutionary continuity to methodological restraint: foundations of comparative psychology

Understanding the rise of animal psychopathology requires first grasping how the scientific study of behaviour and mental life was itself conceptualised, because the assumptions formed in that earlier moment set the terms on which pathological behaviour could later be studied. That formation was not a linear advance toward a unified discipline but a convergence of heterogeneous currents—evolutionary theory, experimental physiology, and philosophical debate about the nature of mind—each approaching behaviour with different assumptions about what it revealed. Together, they established the conceptual tensions that would shape every subsequent controversy about how behaviour should be studied.

Although interest in animal psychology can be traced to antiquity—most notably in Aristotle’s observational writings on reproduction, locomotion, and parental care (Leroi, 2014)—the modern project of comparative psychology took decisive impetus from Charles Darwin (1809–1882). With *The Origin of Species* (Darwin, 1859/2009), mental and emotional traits were framed as products of evolutionary continuity (Mayr, 1982; De Waal, 2016), thereby establishing behaviour as a legitimate site of empirical inquiry rather than an anthropocentric exception. In *The Expression of the Emotions in Man and Animals*, Darwin argued that humans and non-human animals share “fundamental emotions” and capacities for attention, memory, and decision-making, and perhaps even imagination (Darwin, 1872/1998). His three principles of emotional expression—the principle of serviceable associated habits, the principle of antithesis, and the principle of direct action of the nervous system (Ekman, 1992; Fridlund, 1994)—provided not only naturalistic accounts of behaviour but also early attempts to link observable expression with underlying regulatory processes. Even where later authors questioned the universality of basic emotions (e.g., Griffiths, 1997), Darwin’s account positioned behaviour as a window onto internal organisation and made it accessible to scientific analysis.

This evolutionary framing legitimised the comparative study of non-human minds and shaped early work such as George Romanes’s (1848–1894) compilations of anecdotal animal intelligence (Romanes, 1882). Yet Romanes’s reliance on introspection and anthropomorphic interpretation exposed an epistemic tension that would become central to later methodological debates: if behaviour is to serve as evidence of mental organisation, what standards of inference are permissible? Morgan addressed this problem by articulating what became known as Morgan’s Canon (Morgan, 1894), an injunction to prefer simpler psychological explanations unless more complex ones were empirically justified. Far from a narrow methodological dictum, the Canon represented an early attempt to discipline the epistemic relationship between behaviour and inferred internal processes. It fostered a culture of restraint, systematic observation, and controlled experimentation—an important corrective to speculative anthropomorphism.

However, this same commitment to parsimony inaugurated a second tension that would reverberate throughout the twentieth century. The Canon constrained the kinds of complexity that behaviour was allowed to reveal. While it prevented overly mentalistic interpretations, it also laid the groundwork for later reductionist trends that treated behaviour as a minimal “output” of internal mechanisms rather than a complex interface through which regulatory processes could be interrogated. These founding debates—evolutionary continuity against methodological caution, behavioural richness against epistemic parsimony—never resolved into a stable framework. The sections that follow trace how they widened, shaping the experimental turn, the consolidation of method, and ultimately the conditions under which experimental neurosis became thinkable.

## The experimental turn in early comparative psychology

Building on the methodological caution articulated by Morgan and his contemporaries, the early twentieth century witnessed the rising influence of mechanistic approaches in psychology and physiology. These approaches shared a fundamental assumption: behaviour could be explained as the direct consequence of underlying processes, reducible to simple, lawful principles. In practice, however, different researchers interpreted this assumption in divergent ways. Jacques Loeb (1859–1924) exemplified the most radical mechanistic orientation. He viewed animals as reactive systems whose movements were determined by external forces, rejecting “trial and error” learning and the descriptive naturalist style exemplified by Jean-Henri Fabre (1823–1915) as insufficiently rigorous (Pauly, 1987; Fabre, 1905). Loeb formalised his perspective through the concept of tropism, borrowed from plant physiology (de Candolle, 1832), to account for directed movement in response to environmental gradients (Loeb, 1912). His work extended to invertebrates and developmental biology, revealing a vision of behaviour as mechanistically lawful and universally explicable.

Not all early researchers embraced such reductionism. Herbert S. Jennings (1868–1947), studying protozoan behaviour, observed sequences of exploratory actions that could not be captured by rigid tropistic models (Jennings, 1915). By reviving the language of “trial and error,” Jennings stressed rudimentary behavioural flexibility even in unicellular organisms, expanding the conceptual space for understanding behaviour as more than deterministic output. Developmental biologists likewise emphasised organismal complexity in questioning the limits of mechanistic explanation (Haeckel, 1866; Delage, 1895; Hertwig, 1896), while entomological studies of social coordination and cognitive sophistication in insects anticipated later experimental work (Forel, 1904; Wheeler, 1910; Fabre, 1905; Wigglesworth, 1939; Thorpe, 1956).

At the same time, systematic experimental methods began to consolidate. Thorndike introduced puzzle boxes for cats, dogs, and birds, formalising principles of learning such as the laws of effect, use, and disuse and establishing quantitative, reproducible measures (Thorndike, 1898). Yet even his avowedly mechanistic work revealed structured behavioural patterns that hinted at complexity beyond strictly deterministic accounts. A contrasting, ethologically informed current—Köhler’s studies of insight in apes (Köhler, 1917), and the ecologically grounded views of von Frisch, Tinbergen, and the Dutch school of Buytendijk and Bierens de Haan—emphasised behavioural flexibility, species-specific repertoires, and environmental sensitivity, illustrating the coexistence of divergent epistemic commitments within a single field.

The experimental turn thus produced not a consensus but a divided field. Mechanistic models prioritised parsimony, determinacy, and physicochemical causality, treating behaviour as output, while ecological and comparative approaches treated it as structured, flexible, and informative of underlying regulatory processes. That division—unified by a commitment to empirical observation, split over what observation could reveal—set up the debates about variability, individual differences, and stress in which behaviour would increasingly be read as a window onto regulatory dynamics rather than as mere output.

## Methodological consolidation and the roots of animal psychopathology

A wave of experimental innovations in the first decades of the twentieth century expanded both the taxonomic and conceptual scope of comparative psychology, drawing on the growing commitment to methodological rigor. These innovations were not merely technical. They reflected competing assumptions about what behaviour could expose and how it should be examined. Behaviour began to be treated as a source of evidence for patterns of flexibility and context-sensitive adaptations.

Willard S. Small (1870–1943) introduced the maze protocol, providing quantitative measures of learning curves and spatial strategies in rats (Small, 1900). Robert M. Yerkes (1876–1956) extended these experimental approaches to primates, developing multiple-choice tests that linked controlled laboratory psychology with evolutionary and ethological concerns (Yerkes & Yerkes, 1929). Margaret Floy Washburn (1871–1939) synthesised comparative findings across species in *The Animal Mind* (Washburn, 1917), carefully negotiating debates over introspective inference and the criteria for attributing consciousness. Yerkes further contributed early classifications of morphological and functional indicators of “mind,” offering a provisional framework for interpreting behaviour comparatively. These efforts reflect a shared commitment to experimental control, while also exposing divergent epistemic orientations. Some researchers prioritised formal measurement and mechanistic inference, whereas others sought to preserve sensitivity to behavioural complexity, variability, and context.

The consolidation of behaviourism embodied the former orientation. John B. Watson (1878–1958), in his 1913 manifesto *Psychology as the Behaviorist Views It* (Watson, 1913), declared introspection scientifically irrelevant and insisted that observable, reproducible behaviour be treated as the sole legitimate object of psychological inquiry. Watson’s research on sensory discrimination and learning in rats, together with the emotional conditioning experiments involving “Little Albert” (Watson & Rayner, 1920), exemplified a mechanistic, stimulus–response ethos. Developments in neurophysiology simultaneously reinforced this approach. Charles S. Sherrington (1857–1952) elucidated the physiology of reflex arcs and neural inhibition, providing a framework for understanding behavioural integration (Sherrington, 1906), while lesion studies in rats and primates by Franz and Lashley showed the distributed organisation of cognitive functions, challenging strict localisationist assumptions (Franz, 1913; Lashley, 1929). Taken together, these lines of work forged a methodological bridge between controlled behavioural observation and neurophysiological explanation.

Yet as experimental rigour increased, so did the evidence that strictly mechanistic stimulus–response models could not capture the richness, variability, and context-sensitivity of behaviour. Individual differences, taxon-specific adaptations, and complex learning all exposed the limits of reductionism, even as experimental control continued to advance. The questions this left open—what behaviour can reveal, how context-sensitive it is, and how far internal processes may be inferred—were precisely those that the study of behavioural variability, animal psychopathology, and stress would inherit.

## The limits of early mechanistic paradigms: emergence of behavioural variability and complexity

Despite the explanatory power of early mechanistic models, empirical and conceptual limitations increasingly challenged their adequacy. Careful observations of relatively simple organisms revealed behavioural flexibility and context-sensitivity that exceeded the predictive reach of rigid stimulus–response frameworks. Jennings, based on his earlier critiques of tropistic accounts, documented that protozoa and other low-complexity organisms could display escalating or habituating responses to repeated stimulation, patterns incompatible with purely reflexive models (Jennings, 1915). Similarly, studies of phototropism in marine invertebrates showed that internal physiological states, such as limb injury, could shift light-oriented behaviours from direct to lateral movements (Mast, 1906, 1907). William T. Preyer’s (1841–1897) work on starfish *Asterias* revealed graded behavioural sequences in response to irritants—rubbing, shaking, removal attempts, and autotomy—stressing the interplay between external stimuli and internal conditions (Preyer, 1887). Collectively, these findings indicated that even species with limited neural complexity manifested behaviour shaped by prior experience, physiological state, and context, uncovering the limits of strictly mechanistic reductionism.

As comparative psychology matured, attention increasingly turned to genetic and individual variability. Whereas early work sought generalisable, species-typical laws, research with rats—the increasingly dominant experimental model—began documenting stable, heritable differences in learning, sensory discrimination, and emotional reactivity (Hitchcock 1925; Hall 1934a, b; Beach 1950). Selective breeding experiments expanded these findings, showing systematic variation associated with age, sex, and inherited predispositions. Robert C. Tryon (1901–1967) showed that certain traits remained stable across time and contexts, reinforcing the biological significance of individuality (Tryon, 1942). Edward C. Tolman (1886–1959) further argued that intraspecific variation was not merely incidental but epistemologically essential for understanding purposive behaviour (Tolman, 1932, 1938). These findings echoed that behaviour could not be fully captured by averaging across populations or reducing actions to simple stimulus–response chains.

The resulting conceptual challenge demanded theoretical frameworks capable of integrating external stimuli, internal states, and enduring behavioural tendencies. Behaviourists in the Watsonian tradition often treated individual differences as residual noise from learning histories, while ethologists like Konrad Lorenz (1903–1989) acknowledged variability but interpreted it primarily through species-typical patterns (Lorenz, 1937). Formalised mechanistic approaches, represented by Loeb, Watson, Pavlov, and Thorpe, sought to reconstruct complex behaviour from elementary units—reflexes, tropisms, and conditioned associations—grounding explanations in physics and physiology. In contrast, Tolman’s purposive behaviourism (Tolman, 1932, 1948) and Clark L. Hull’s (1884–1952) drive-reduction theory (Hull, 1943) introduced internal variables, goals, and probabilistic outcomes, extending mechanistic reasoning towards the regulation of behaviour without losing experimental precision.

Yet even these sophisticated theories fell short of capturing the full repertoire and context-sensitivity of naturalistic behaviour. Strategic change, emergent dynamics, and nonlinear interactions persisted as conceptual and methodological challenges, revealing the inadequacy of strictly reductionist paradigms. This growing awareness catalysed methodological innovations capable of rendering such interactions observable. Among these, Ivan Pavlov’s systematic study of conditioned reflexes proved particularly transformative. By formalising stimulus–response associations within physiological frameworks, Pavlov bridged psychology and neurobiology, opening experimental pathways for investigating learning, emotion, and stress in a controlled, measurable way. The period’s defining tensions—parsimony against behavioural richness, reduction against systemic complexity, the general law against the individual case—were thus not resolved but bequeathed to the programmes that followed.

## Early experimental approaches to learning and behavioural modification

Early comparative psychology increasingly focused on learning as a process of adaptive change, reflecting a growing recognition that behaviour is shaped by experience and context. Learning was understood as modifications in behavioural responses arising from interactions with the environment, often guided by considerations of efficiency, self-preservation, or goal achievement. Crucially, “experience” did not imply introspective awareness but referred to the cumulative influence of prior interactions on future action (Heron, 1949). From this perspective, learning offered a natural window onto the internal regulatory processes that generate flexible, context-sensitive behaviour.

Early attempts to formalise behavioural adaptation exposed both its potential regularities and its conceptual ambiguities. The so-called “law of least effort” described the tendency of animals, such as rats navigating mazes, to adopt the shortest or most energetically efficient path to a goal (Waters, 1937). While descriptive, this principle raised foundational questions: how should effort, motivation, or goal-directedness be operationalised? How could researchers distinguish true learning from transient changes due to fatigue or sensory adaptation? These ambiguities accentuated the broader challenge of capturing the richness of behaviour in systematic experimental terms. Empirical work across diverse species reinforced the idea that even “simple” organisms could show meaningful behavioural plasticity. Repeated stimulation of slugs (*Limax maximus*) produced shifts from withdrawal to exploratory responses, indicative of habituation (Frandsen, 1901). Honeybees revealed discrimination learning between colours and shapes, assigning predictive value to stimuli (von Frisch, 1914). Such findings showed that learning was neither a uniform process nor reducible to immediate stimulus–response chains. Instead, it reflected the dynamic interplay between organism, environment, and prior experience.

These phenomena were increasingly studied through paradigms designed to quantify learning under controlled conditions—the same puzzle boxes and mazes introduced above, now turned explicitly to measurement. Thorndike’s boxes produced replicable learning curves across repeated trials (Thorndike, 1898), and Small’s mazes enabled systematic assessment of orientation, memory, and spatial problem-solving (Small, 1900). The gain in reproducibility came at a price: by constraining behaviour to standardised tasks, such methods risked reducing complex processes to simplified proxies and obscuring the richness and flexibility of natural behaviour.

Experimental work further explored the physiological and motivational determinants of learning. Internal drives such as hunger, fear, or reproductive states were identified as modulators of behavioural performance, distinguishing them from external incentives that shaped goal-directed action (Harlow, 1949; Heron, 1949). Controlled devices such as operant conditioning chambers (Skinner, 1938) and the Columbia obstruction apparatus (Warden & Aylesworth, 1927; Thorpe, 1956) enabled precise assessment of responses to environmental contingencies. One early, and often overlooked, line of inquiry examined hormonal influences—for example on maternal and excretory behaviours in dogs—suggesting that behaviour could serve as a measurable interface with internal regulatory systems (Martins & Valle, 1948).

These paradigms advanced experimental control, albeit at a recurring cost. By privileging replicable metrics and discrete response units, they narrowed the very richness, variability, and context-sensitivity that later accounts of regulation and its breakdown would require. It was against this backdrop that Pavlov’s conditioned-reflex programme emerged. In linking stimuli to physiological and behavioural responses, Pavlov framed behaviour as an observable interface with underlying regulatory processes. Rather than reducing behaviour to mechanism, his programme exposed the difficulty of capturing its core features within experimental frameworks—precisely the challenge that the study of experimental neurosis would later confront.

## Pavlovian conditioning and the emergence of experimental neurosis

That programme repays closer attention, for it was here that those conditions first arose. Originally focused on the neural control of digestion, Pavlov’s research at the Institute of Experimental Medicine in St. Petersburg shifted toward salivary reflexes. Dogs learned to anticipate food when neutral stimuli were repeatedly paired with feeding. This learned response, the “psychic reflex,” became the prototype of conditioned reflexes and a cornerstone for subsequent psychological and physiological theories (Pavlov, 1928; Todes, 2014). Pavlov’s work paralleled the identification of hormonal regulation by William Bayliss (1860–1924) and Ernest Starling (1866–1927). Their discovery of secretin (Bayliss & Starling, 1902) was followed by Starling’s introduction of the term “hormone” to name this newly recognised class of blood-borne chemical messengers (Starling, 1905). Together, these advances helped establish that behaviour and physiology are coordinated by two distinct but interacting control systems—rapid, spatially targeted signalling through the nervous system, and slower, diffuse signalling by hormones circulating in the blood.

Pavlov distinguished between unconditioned reflexes—automatic responses mediated by fixed neural pathways—and conditioned reflexes, which are acquired through repeated pairings of neutral and biologically significant stimuli. Crucially, conditioned responses—such as a dog salivating to the sound of a metronome—were not treated as simple stimulus–response links but as expressions of dynamic neural regulation (Gantt, 1944). Their fragility was central to this view. Conditioned responses could be disrupted by distracting stimuli (“external inhibition”), altered through changes in temporal relations (“internal inhibition”), or overridden by competing associations. Pavlov further articulated a hierarchical organisation of inhibitory processes, including the “inhibition of inhibition,” showing that suppression itself was subject to regulation. Together, these principles formed the basis of his theory of higher nervous activity, which held that learning reorganises neural function through dynamic and reversible processes rather than permanent anatomical change (Pavlov, 1928, 1941).

In the early 1920s, Pavlov and Maria K. Petrova (1874–1948) explored how sustained conflict affected conditioned behaviour. Working with dogs of differing temperaments—one calm, one excitable—they observed that reactive animals struggled with tasks requiring delayed responses or discrimination, often showing agitation or behavioural shutdown (Petrova, 1924, 1926). These breakdowns were interpreted as unresolved conflicts between neural excitation and inhibition (“collisions”) representing failures of regulatory control rather than mere errors (Pavlov, 1941; Windholz & Grimsley, 1992). Pavlov termed these states experimental *neurosis*, attributing their origin to physiological disequilibrium under sustained conflict, a concept likely inspired by a case in *Studies on Hysteria* (Breuer and Freud, 1895/1999) (Todes, 2014). By intensifying ambiguity in the conditioned stimulus, Pavlov could induce experimental neurosis in some animals, particularly those with excitable temperaments. This state was a genuine physiological and behavioural disruption, not a metaphorical concept, providing a controlled model for maladaptive responses and behavioural pathology. Pavlov’s experiments thus extended reflex theory into the domain of mental disorders, linking laboratory learning paradigms to broader conceptions of stress-related dysfunction.

Eventually, these findings resonated with emerging comparative psychology internationally (Ruiz et al., 2003). Unlike contemporaries who focused on adaptive performance and measurable outputs, Pavlov revealed the fragility of behavioural regulation under sustained demand. His conflict protocols anticipated later approaches to the study of anxiety, trauma, and emotional dysregulation. Exemplifying how prolonged tension and irresolvable demands could destabilise behavioural control, the work stressed the importance of internal states, motivation, and physiology in modulating learning outcomes, laying the groundwork for integrating endocrine mechanisms and refined experimental tools (Thorpe, 1956). Conceptually, Pavlov recast mental pathology as a consequence of limits in physiological regulation. Behavioural collapse was no longer seen as solely the result of external disruption, but as an intrinsic failure of regulatory systems under sustained conflict. This reconceptualisation shaped later research, particularly in the United States, where Pavlovian principles influenced psychodynamic, developmental, and longitudinal approaches to maladaptation. Experimental neurosis thus became a conceptual bridge linking behaviourism, psychiatry, and endocrinology, establishing stress vulnerability as a central theme in animal models of psychopathology.

## Expanding the experimental neurosis paradigm: from Pavlov to pasture

Advancing beyond Pavlov’s experimental neurosis framework, early- to mid-twentieth-century US-based researchers reshaped the paradigm through innovations in species selection, environmental design, and conceptual orientation. Figures such as Liddell at Cornell and Masserman at the University of Chicago reoriented the study of behavioural pathology—understood here as seemingly maladaptive responses—towards contexts that would make visible the complex interplay between conditioned reflexes, emotional states, and social environments. These developments marked a transition in how behaviour itself was conceptualised. Behaviour became understood as a primary site of interrogation, a level of organisation through which dysregulation could be identified, experimentally manipulated, and compared across species. This shift laid groundwork for comparative psychiatric analyses by showing that behavioural disturbances could be systematically produced, stabilised, and studied in laboratory animals (Barlow et al., 2014; Kirk & Ramsden, 2018; Koch, 2019).

Masserman, a psychiatrist influenced by both Pavlovian methodology and psychoanalytic theory, developed experimental paradigms using cats rather than dogs. In the 1940s, he argued that cats showed complex and subtle behavioural repertoires, particularly under conditions designed to elicit what he termed motivational conflict—a notion that was in line with broader psychoanalytic conceptions of conflicting drives. His procedures combined food deprivation with aversive stimuli (air blasts or electric shocks) delivered as cats approached a food box, thereby creating situations in which positive and negative motivations were placed in opposition. The resulting displays—withdrawal, inhibited grooming, refusal to approach the food box even when hungry—were interpreted as feline analogues of neurotic symptoms and, more specifically, as experimentally induced counterparts to clinical conflict states observed in human patients. Documented through sequential photography and detailed narrative accounts, these experiments were presented in a manner that closely resembled psychiatric case studies rendered in non-human form (Masserman, 1943b).

Masserman’s approach differed from Pavlov’s not only in species but in his explicit integration of psychoanalytic concepts. He framed experimental neurosis in terms that resonated with contemporary psychoanalytic theory, rather than focusing solely on cortical excitation-inhibition balance. Neurosis arose in part from internal motivational conflict between drives (hunger, fear), rather than primarily from simple failures of stimulus discrimination. This represented not an abandonment of Pavlovian methods but an attempt to provide those conditioning procedures with a psychodynamic interpretation, using laboratory conflict paradigms to model mechanisms that psychoanalysts had inferred from clinical observation. In this framework, the pathological state emerged when the organism could not resolve competing internal demands under conditions that made both gratification and avoidance impossible (Masserman, 1943a; Winter, 2016).

Parallel but distinct developments took place in Liddell’s laboratory at Cornell. Confronted with the practical and ethical limitations of canine models—and, as Kirk and Ramsden (2018) note, motivated by the logistical advantages of working with livestock already housed on the farm—Liddell adopted sheep and goats as experimental organisms (Liddell, 1934; Liddell et al., 1936; Kirk & Ramsden, 2018). Though initially uncooperative with salivary reflex methods, these animals responded robustly to conditioning protocols involving auditory cues and mild electric shocks to the foreleg. More importantly, they showed a rich array of motor and social behaviours—approach and avoidance, freezing, vocalisation, altered flock dynamics—that could be meaningfully tracked both inside the experimental chamber and in their barn and pasture environments (Anderson & Liddell, 1935; Anderson & Parmenter, 1941).

Liddell’s most significant methodological innovation was the construction of what he called a “mimic farm”, a hybrid research environment that combined laboratory control with elements of the animals’ natural social habitat. Animals were studied both in controlled indoor conditioning chambers and in barns, pastures, and social groups. This design allowed continuous tracking of behavioural and physiological markers over extended periods, offering a genuinely longitudinal perspective on inner states and their persistence, an approach that anticipated later chronic-stress animal models. Autonomic measures (e.g., heart rate, respiration), kymographic traces, and detailed behavioural observations were brought together to produce layered profiles of emotional and regulatory change across months and, in some cases, years (Liddell, 1947). This represented a pragmatic adaptation to persistent challenges. Symptoms that appeared during conditioning sessions often proved transient or inconsistent unless the animals’ behaviour outside the laboratory was also monitored. By observing ‘neurotic’ sheep in their flock, Liddell could reveal that laboratory-induced disturbances were not merely situational responses but enduring alterations in social behaviour and autonomic regulation.

A key finding from Liddell’s work was the persistence of behavioural abnormalities long after conditioning ceased. Sheep that had experienced prolonged difficult discrimination tasks often showed chronic hypervigilance, altered social dynamics, excessive vocalisation, and emotional volatility. These symptoms resisted extinction, sometimes persisting for months or years, indicating a shift from transient conditioned responses to more entrenched patterns of dysregulation (Liddell, 1947). What emerged was not merely an experimental artefact but a stable state of maladaptation, a phenomenon that anticipated later concepts such as chronic stress, allostatic load, and affective pathology (McEwen & Stellar, 1993; McEwen, 2007).

Importantly, both Masserman’s and Liddell’s work expanded the conceptual terrain of comparative psychopathology by treating behaviour itself as a primary site of inquiry. Behaviour was no longer a secondary readout of neural function but a complex, autonomous system through which dysregulation could be identified, modelled, and studied across species (Masserman, 1946; Liddell, 1947). Yet their approaches diverged significantly in both method and interpretation. Masserman, working within tightly controlled chambers, stressed how experimentally induced conflict could produce behavioural disturbances that persisted beyond the immediate conditioning session, an idea central to his argument that neurotic symptoms reflected enduring motivational conflict rather than momentary failures of discrimination (Masserman, 1946). Liddell, by contrast, increasingly departed from Pavlovian orthodoxy. He rejected Pavlov’s physiological explanation of neurosis (the “clash” of excitation and inhibition) and instead stressed the emotional and social dimensions of conditioning (Liddell, 1947). His turn toward ethological observation—studying animals in semi-naturalistic social groups—represented not a refinement of Pavlovian theory but a move that enabled a broader understanding of behaviour in ecological and social context.

Together, Masserman’s and Liddell’s innovations reflected important but divergent responses to the challenges of studying behavioural pathology experimentally (Kirk & Ramsden, 2018; Koch, 2019). While Masserman sought to provide experimental validation for psychoanalytic concepts of internal conflict, Liddell increasingly moved towards an ethological perspective that emphasised the animal’s total life situation, including its social relationships, developmental history, and environment beyond the laboratory. Rather than representing a unified advance, their work embodied fundamental tensions about what level of analysis—whether physiological reflex, learned association, motivational conflict, emotional regulation, or social adaptation—could most fruitfully illuminate the nature of neurosis. Crucially, these tensions were not only disputes between research traditions but also persisted within the experimental neurosis paradigm itself, where researchers interpreted similar behavioural disturbances through incompatible theoretical lenses. These debates mirrored broader disagreements within psychiatry over whether neurosis should be understood as cortical dysfunction (Pavlov), learned maladaptation (behaviourism), unconscious conflict (psychoanalysis), or failures of biopsychosocial adjustment (Meyer’s psychobiology). Rather than resolving these controversies, the experimental neurosis paradigm became a point of convergence where competing frameworks intersected, each claiming behaviour as the key interface through which their preferred level of explanation might be validated (LeDoux, 2000; Blanchard & Blanchard, 2008). A third US-based programme, developed by W. Horsley Gantt at Johns Hopkins, extended the paradigm along yet another axis. Working under Adolf Meyer (1866–1950) while retaining Pavlov’s dogs as subjects, Gantt shifted the analytic emphasis from the individual conditioning session to the long-term trajectory of the animal. His method centred on detailed longitudinal life charts that followed single dogs across years, integrating conditioning histories, emotional reactions, social relationships, and physiological states into a continuous behavioural record (Gantt, 1944; Ramsden, 2018). Where Masserman manipulated motivational conflict within the chamber and Liddell extended observation outward into the flock, Gantt extended it across time, treating the persistence and evolution of disturbance in the individual as the primary object of study. These unresolved tensions laid the groundwork for subsequent conceptual reframing.

## Redefining neurosis: from disorder to dysregulation

Following from these mid-century developments, the conceptualisation of neurosis underwent significant evolution, though not along a single, unified trajectory. The field fractured simultaneously along conceptual (conflict vs. adaptation vs. reflex imbalance), methodological (strict chamber protocols vs. semi-naturalistic observation), and interpretive (psychoanalytic, Pavlovian, behaviourist, psychobiological) lines. Divergent programmes—Masserman’s conflict chambers, Liddell’s mimic farm, Gantt’s longitudinal case studies—reflected not theoretical consensus but pragmatic attempts to address the difficulties of modelling behavioural pathology experimentally. Yet these approaches shared a fundamental reorientation. Neurosis came to be viewed less as a discrete disorder with a singular etiology and more as a multilevel failure of adaptive regulation spanning behavioural, emotional, and physiological systems. This shift was gradual and uneven, but it nevertheless marked an important transition in the status of behaviour itself. From symptom to system, from mere output to a primary interface through which dysregulation could be inferred.

The term neurosis has historically carried shifting meanings. Originating in the eighteenth century as a label for nervous system disorders without anatomical lesions (Cullen, 1793), by the early twentieth century it had come to signify a broad range of functional psychological disturbances, such as phobias, anxiety states, and obsessional conditions. These meanings continued to evolve. Pavlov’s experimental work reframed neurosis within a conditioning context, interpreting it as a breakdown in stimulus perception rooted in cortical imbalance between excitation and inhibition (Pavlov, 1928). Yet this interpretation was far from universally accepted. Psychoanalytically oriented psychiatrists, following Freud, understood neurosis as arising from unconscious intrapsychic conflict, a formulation that had never conceptualised neurosis as a discrete, unitary disorder but instead as a dynamic process involving multiple levels of psychological functioning (Freud, 1926/1959). As Koch (2019) has shown, the concept’s definitional flexibility was a persistent feature of its clinical and experimental life. By the time US-based experimental researchers took up the topic in the 1920s and 1930s, the view that neurosis reflected complex systemic processes rather than simple deficits was already well established in clinical psychiatry (Lamb, 2014).

What researchers like Liddell, Masserman, and Gantt attempted, therefore, was not to create a systemic understanding of neurosis but to integrate existing psychodynamic concepts with Pavlovian experimental methods. Influenced directly or indirectly by Meyer, whose psychobiology movement explicitly sought to bridge physiological, psychological, and social perspectives, they aimed to provide an experimental grounding for clinical theories that were simultaneously evolving in psychiatric practice, thereby lending those theories a level of empirical credibility that purely clinical observation could not secure (Meyer, 1908/1950; Masserman, 1944). Masserman’s conceptual framework was further informed by Kurt Goldstein’s (1878–1965) organismic theory, which understood neurological and behavioural breakdown not as localised deficits but as failures of the organism to maintain coherent function under environmental demands exceeding adaptive capacity—a perspective Masserman explicitly invoked in characterising experimental neurosis as systemic dysregulation rather than discrete symptom (Goldstein, 1934/1995; Masserman 1943a). Their contribution lay not in theoretical innovation but in methodological synthesis. In doing so, they showed that conditioning procedures could produce behavioural disturbances that resembled, and might help elucidate, the complex, persistent symptoms observed in human patients.

This experimental synthesis revealed patterns of dysregulation that transcended simple reflex disruption. Across different laboratories and species, researchers documented a cluster of behavioural and autonomic disturbances—including emotional lability, heightened arousal, social withdrawal, and resistance to extinction—that more closely resembled clinical descriptions of anxiety and neurotic conditions than Pavlov’s original accounts of discrimination breakdown (Cannon, 1932). The acceptance of such variability and complexity as meaningful marked a methodological departure from earlier rigid stimulus–response models towards approaches that incorporated longitudinal observation, individual case histories, and attention to behaviour across multiple contexts (Masserman, 1944; Gantt, 1944). Liddell’s mimic farm exemplified this shift. By tracking animals from the conditioning chamber to the barn to the pasture, his group showed that experimentally derived disturbances constituted systemic alterations in behavioural regulation rather than isolated conditioned responses.

Physiological studies complemented and extended these behavioural observations. Attention shifted to the role of endocrine systems in sustaining dysregulation. Although Hans Selye’s (1907–1982) original formulation of stress emerged from general physiology rather than psychiatry, his work showed that stress episodes could induce persistent biochemical changes that compromised neural stability and behavioural flexibility, supporting the idea that neurosis involved chronic alterations in homeostatic regulation rather than isolated reflex failures (Selye, 1936, 1946, 1950; Mason, 1971, 1972). This physiological research resonated with, and provided mechanistic grounding for, the behavioural findings, reinforcing the view that neurosis represented a multilevel disorder of dysregulation rather than a disturbance confined to any single system or level of analysis.

These experimental developments reflected and paralleled, rather than initiated, the evolving relationship between animal research and human psychiatry in mid-century United States. Under Meyer’s leadership at Johns Hopkins, the psychobiology movement had already established institutional frameworks for integrating physiological, psychological, and social perspectives, with particular emphasis on life-charting, habit formation, and the patient’s total life context. Gantt worked directly under Meyer; Masserman trained at the Phipps Clinic; and Liddell—though based at Cornell—corresponded extensively with Meyer, Gantt, and visiting psychiatrists. The relationship between experimental findings and clinical theory was often reciprocal, or even inverted, compared to a simple model in which “basic research informs clinical practice.” Clinical observations of war neuroses, for instance, prompted experimental investigations. Psychoanalytic concepts of conflict informed the design of conditioning experiments. And ethological observations of disrupted maternal–infant bonding in Liddell’s flocks resonated with concurrent clinical interest in attachment and early experience (Bowlby, 1951). The experimental neurosis paradigm thus embodied ongoing tensions within mid-century psychiatry about whether mental disorders were best understood through physiological, behavioural, or psychodynamic frameworks—tensions that would shape the field’s trajectory in subsequent decades.

This conceptual evolution—or perhaps more accurately, this proliferation of competing frameworks using behaviour as a common observational interface—positioned neurosis as a heuristic bridge connecting physiological processes, behavioural outcomes, and environmental stressors across species. Neurosis ceased to function primarily as a strict diagnostic category and instead served as a comparative construct. A way of thinking about dysregulation that could accommodate evidence from conditioning experiments, clinical observations, and physiological measurements without requiring full theoretical integration. While psychodynamic traditions emphasised unconscious conflict, the mid-century experimental framework foregrounded dynamic failures in system-level behavioural regulation. An idea that, while compatible with aspects of psychoanalytic theory, could be articulated in the language of physiology, learning theory, or systems biology. This emphasis on regulation and dysregulation as organising concepts would prove influential. It resonates with contemporary views of psychopathology that stress dysregulation as a transdiagnostic feature cutting across traditional diagnostic boundaries (Beauchaine & Cicchetti, 2019; Insel et al., 2010) and require multi-level explanation integrating molecular, neural, behavioural, and social processes (Kendler, 2012).

However, this apparent convergence around concepts of regulation and dysregulation should not obscure the fundamental theoretical tensions that persisted—and that would soon contribute to a major upheaval in psychiatric classification. The term neurosis itself became increasingly contested in the 1960s and 1970s, caught between incompatible explanatory frameworks. Was it a physiological disorder, a learned maladaptation, an unconscious conflict, or a social/interpersonal problem? The neo-Kraepelinian turn in American psychiatry during this period emphasised atheoretical, descriptive diagnosis focused on observable symptoms rather than presumed etiologies. This movement, which culminated in the DSM-III (1980), led to the removal of neurosis as a superordinate diagnostic category from official psychiatric nomenclature (Decker, 2013). The specific anxiety, mood, and somatic symptom disorders that had been grouped under neurosis were reclassified as distinct entities, and the term itself—freighted with psychoanalytic commitments—was abandoned in favour of putatively theory-neutral descriptive categories. In this shift, the experimental neurosis paradigm was increasingly marginalised, its multilevel model of behavioural dysregulation incompatible with the narrower symptom-based framework that now defined psychiatric classification.

Yet the foundational contributions of experimental neurosis research persisted despite this nosological rupture. The recognition that behavioural pathology often involves multilevel dysregulation—that symptoms reflect not discrete lesions but systemic failures of adaptation—continues to inform contemporary approaches to psychopathology. Current frameworks conceptualise mental disorders not as static disease entities but as dynamic disruptions in the organism’s capacity to coordinate and regulate competing internal and external demands (Beauchaine & Cicchetti, 2019). The shift from thinking about neurosis as a thing (a disorder one has) to a process (a failure of regulation) reflects the lasting influence of mid-century experimental work that took behaviour seriously—not as a mere symptom to be explained away by underlying causes but as a complex system whose organisation and breakdown could reveal the nature of psychopathology. In this sense, the experimental neurosis paradigm’s most enduring contribution is methodological rather than theoretical. It established behaviour as a legitimate level of analysis and showed that behavioural dysregulation could be systematically studied, an understanding that continues to shape contemporary work on chronic stress, allostatic load, and resilience. Examining how this methodological framework emerged from the interplay between laboratory research and clinical psychiatry reveals what that interplay implies about the status of behaviour within psychopathological knowledge.

## Experimental neurosis and clinical psychiatry: the reciprocal construction of psychopathological knowledge

The mid-century expansion of experimental neurosis research occurred not in isolation from clinical psychiatry but in continuous dialogue with it, a dialogue that was neither unidirectional nor straightforward. As discussed above, the relationship between laboratory findings and clinical theory was often reciprocal or even reversed from what a simple model of “basic research informing practice” would suggest. Clinical observations prompted experimental designs; psychoanalytic concepts shaped conditioning protocols; and ethological findings from animal colonies resonated with emerging clinical theories of attachment and early experience. Experimental neurosis functioned as a bidirectional interface between laboratory and clinic, revealing not only how animal models informed psychiatric thinking but also how clinical frameworks structured what researchers could see, measure, and interpret in their animals.

The notion that animal behaviour could illuminate human psychopathology was contested from the outset. Sigmund Freud (1856–1939) had famously declared neurosis to be a “human privilege,” arguing that the unconscious conflicts underlying neurotic symptoms required symbolic capacities unavailable to non-human animals (Freud, 1926/1959). Psychoanalytically oriented clinicians in the 1920s and 1930s thus viewed experimental neurosis research with considerable scepticism, questioning whether conditioned reflex breakdowns bore any meaningful relationship to the intrapsychic dynamics they encountered in clinical practice. Yet this scepticism was not universal. Figures like Masserman explicitly sought to bridge Pavlovian and Freudian frameworks, arguing that conditioning procedures could provide experimental access to conflict mechanisms that psychoanalysts had inferred from verbal reports and dream interpretation. His work with cats, designed to create situations of motivational conflict analogous to those posited by psychoanalytic theory, represented an attempt to render psychodynamic concepts experimentally tractable (Masserman, 1943a).

Gantt’s longitudinal method, introduced above, carried a specifically psychobiological conviction. That is, that mental disorders should be understood through integrated attention to physiological, psychological, and social factors. His life charts for individual dogs directly paralleled Meyer’s biographical approach to human patients (Gantt, 1944; Ramsden, 2018). This was not mere methodological borrowing. It reflected a deeper commitment: that behaviour, observed systematically over time and across contexts, provides access to regulatory organisation that cannot be inferred from physiological measures or verbal reports alone. In this sense, Gantt’s dogs functioned not as simplified models of human patients but as organisms whose behavioural dynamics could be studied with a completeness and control unavailable in clinical settings.

The relationship between experimental models and clinical syndromes remained ambiguous, however. Pavlov likened the behavioural collapse he observed in dogs subjected to difficult discriminations to neurasthenia, a diagnosis prevalent in early twentieth-century psychiatry that focused on exhaustion, irritability, and autonomic instability (Pavlov, 1941). Liddell drew parallels between the chronic hypervigilance of his neurotic sheep and both anxiety states and neurocirculatory asthenia, a condition observed in soldiers during World War I characterised by cardiovascular symptoms arising under the strain of combat (Liddell, 1944). These analogies were cautious and provisional. Neither Pavlov nor Liddell claimed that animal neuroses were identical to human psychiatric conditions. Rather, they suggested that shared regulatory mechanisms, particularly the failure of inhibitory control under sustained demand, might underlie behavioural breakdowns across species. This functional or mechanistic analogy differed fundamentally from the diagnostic thinking that dominated clinical psychiatry. Where clinicians organised symptoms into discrete categories (neurasthenia, anxiety neurosis, obsessional neurosis), experimental researchers increasingly focused on dysregulation as a dynamic process that could manifest in variable symptom profiles depending on species, individual temperament, and environmental context. This emphasis on regulation and dysregulation positioned experimental neurosis as a heuristic construct rather than a strict disease model, a framework for thinking about how adaptive systems break down under chronic stress, rather than a one-to-one mapping of animal behaviours onto human diagnostic categories.

The translational ambitions of experimental neurosis research were nevertheless real and consequential. During World War II, observations of combat stress and traumatic neurosis prompted renewed interest in animal models. Gantt devoted an entire chapter of his 1944 monograph to “natural emotional shocks; traumatic and experimental war neuroses,” explicitly linking laboratory findings to the psychiatric casualties observed in returning soldiers (Gantt, 1944; Ramsden, 2018). Masserman similarly invoked wartime experiences to justify his work on conflict-induced pathology. Yet as Koch (2019) documents, attempts to experimentally recreate traumatic conditions—exposing dogs to loud noises, explosions, or other frightening stimuli—often failed to produce the persistent symptoms associated with difficult discrimination tasks. This finding was theoretically significant. It suggested that the chronic, unresolvable nature of conflict—rather than the intensity of a single aversive experience—was the critical factor in producing stable neurotic states. Trauma, in this framework, was reconceptualised not as a discrete shock but as a condition of prolonged regulatory demand without resolution. This view anticipated key features of mid-century stress research, including Selye’s elaboration of the General Adaptation Syndrome (Selye, 1950) and later work on learned helplessness (Maier & Seligman, 1976). In retrospect, the most important contribution of experimental neurosis research to clinical psychiatry may not have been the identification of specific disease mechanisms but rather the demonstration that behavioural pathology could be experimentally produced in normal, previously healthy organisms. This challenged prevailing clinical assumptions that neurosis arose primarily from constitutional predispositions or early developmental trauma. By showing that chronic conflict alone—in the absence of pre-existing vulnerability—could induce lasting behavioural and autonomic disturbances, experimental work reframed neurosis as a potential outcome of environmental demands exceeding adaptive capacity, rather than as an expression of underlying weakness or pathology.

Yet this very success created new problems. If neurosis could be produced experimentally in any animal subjected to sufficiently demanding conditions, what distinguished pathological from normal stress responses? How should researchers define the boundary between adaptive strain and maladaptive breakdown? These questions became increasingly urgent as the experimental neurosis paradigm expanded. They also reflected deeper unresolved tensions within psychiatry itself. Was neurosis a discrete disease entity, a dimensional trait, or a functional concept describing regulatory failure? The inability to resolve this question contributed to neurosis’s eventual removal from psychiatric nomenclature, a transformation that reflected deeper conceptual tensions the experimental paradigm could not resolve. Importantly, however, the conceptual legacy of experimental neurosis persisted in altered form. The framework of dysregulation, chronic stress, and loss of adaptive flexibility that emerged from mid-century animal research continued to inform psychiatric thinking even after the term “neurosis” was abandoned. Contemporary concepts such as allostatic load (McEwen & Stellar, 1993), stress sensitisation, and transdiagnostic models of anxiety and mood disorders (Beauchaine & Cicchetti, 2019) can be traced, in part, to the regulatory models developed through experimental neurosis research. More fundamentally, the experimental tradition established a methodological precedent. Behaviour, systematically observed under controlled yet ecologically valid conditions, provides essential access to regulatory dynamics that cannot be inferred from physiology alone. This principle, although contested and at times obscured by reductionist trends, remains central to comparative psychiatry and translational neuroscience.

The relationship between experimental neurosis and clinical psychiatry thus exemplifies a broader pattern in the history of psychopathology: the co-construction of laboratory and clinical knowledge through iterative, bidirectional exchange. Animal models did not simply validate or refute clinical theories. They transformed the terms in which psychopathology could be conceptualised, shifting attention from static disease categories to dynamic processes of adaptation and breakdown. At the same time, clinical frameworks structured what experimenters could recognise as pathological, how they interpreted behavioural variability, and what mechanistic explanations seemed plausible. In this sense, experimental neurosis functioned not as a bridge from laboratory to clinic but as a shared conceptual space—a site where competing understandings of behaviour, regulation, and pathology were negotiated, contested, and provisionally reconciled. Understanding this history is essential for contemporary efforts to develop translatable models of mental disorder, as it reveals both the possibilities and the limitations inherent in using animal behaviour to access human psychopathology.

## Epistemological lessons: behaviour as irreducible interface in mechanistic explanation

The historical trajectory outlined above reveals a persistent conceptual challenge in scientific explanation: how should the relationship between behaviour and mechanism be understood? This question is not merely methodological but fundamentally epistemological. It concerns what can be known, through what means, and at what level of analysis. The early comparative psychopathology tradition, particularly the experimental neurosis paradigm, articulated a position that remains both relevant and contested. Behaviour is not a derivative output of neural mechanisms, but the primary interface through which regulatory organisation becomes scientifically accessible. Mechanistic reduction that subordinates behaviour to underlying processes does not simplify explanation; rather, it eliminates the very phenomenon that renders those processes intelligible as organised, functional systems.

The argument that behaviour constitutes the primary interface of regulatory organisation can be stated more precisely. Living systems show integration, context-sensitivity, developmental plasticity, and adaptive flexibility, properties that, as general systems theory has long recognised (von Bertalanffy, 1968; Wiener, 1948; Weiss, 1969), emerge from but cannot be fully predicted by the characteristics of individual components. As Ernst Mayr (1904–2005) argued compellingly, biological explanation must account for organisation across multiple levels, each with its own dynamics, and specify how activity at one level generates structured phenomena at another (Mayr, 1982). Cybernetic theories of feedback regulation and homeostatic control (Wiener, 1948; Ashby, 1952) provided formal frameworks for conceptualising adaptive systems and their breakdown, though these developed largely independently of the experimental neurosis tradition. The locus of integration was itself relocated repeatedly across this period—from the integrative action of the nervous system (Sherrington, 1906), to the whole organism, to the biographical individual of Meyer’s psychobiology, and finally to the formal feedback loop of cybernetics (Wiener, 1948; Ashby, 1952)—so that the changing status of behaviour was accompanied by an equally consequential, and arguably more discontinuous, shift in what integration was taken to be and how it could be studied. That history lies beyond the present scope and merits separate treatment. This is the challenge of mechanistic integration (Bechtel, 2008; Craver, 2008): relating molecular, cellular, circuit, and systems-level processes to the organised patterns of behaviour through which organisms adapt to their environments. Because the meanings proper to the behavioural level do not survive translation downward, the task is to relate these levels, not to reduce the higher to the lower. Contemporary psychiatric theory recognises that mental disorders require explanation at multiple levels simultaneously, with no single level—genetic, neural, or behavioural—being causally or explanatorily more fundamental than the others (Kendler, 2012). The primacy defended here is of a different kind and is fully compatible with that view. It is not a claim that behaviour is a more fundamental level of being, but a claim about functional significance. Whether a given neural process counts as adaptive regulation, as learning, or as pathology is fixed by its role in behaviour. The relation between the levels is in this respect asymmetric. The organisation of behaviour can be characterised without first specifying the mechanism that realises it, whereas the functional significance of that mechanism cannot be established without reference to the behaviour it serves. Unlike neural activity, which must be accessed indirectly and interpreted through theoretical frameworks, behaviour is directly observable as the organism’s engagement with its environment. More critically, it is at the level of behaviour that regulatory processes acquire functional meaning. A pattern of neural activity becomes a “fear response,” a “decision,” or a “coping strategy” only by virtue of the context in which it occurs and the consequences it produces. As Steven P. R. Rose (1938–2025) argued, “each level of organisation has its own meanings, which disappear at lower levels” (Rose, 1997). Reducing behaviour to mechanism thus entails a loss of explanatory content, erasing the functional significance that gives neurophysiological activity its meaning. A reductionist might object that this loss is only apparent—that functional significance is deferred rather than eliminated, recoverable once the underlying mechanism is fully specified. The historical record tells against that reassurance. The categories that make a pattern of activity a coping strategy or a failure of inhibition are fixed by behavioural context and consequence. A description that brackets those categories does not postpone their recovery so much as discard the criteria by which recovery could be recognised. This is not a claim about every behaviour. The function of a simple reflex may be legible in its mechanism. But wherever functional significance is constituted relationally—by context, history, and consequence—it cannot be recovered from a description confined to the mechanism, since those relational facts are excluded by the descriptive frame rather than merely absent from it. The richer and more context-dependent the behaviour, the more decisively this holds.

The early experimental neurosis tradition put this principle—the epistemic and functional primacy of behaviour—into practice. Researchers such as Liddell, Gantt, and Masserman did not treat behaviour as a mere dependent variable to be correlated with physiological measures. Instead, they treated it as a structured, dynamic phenomenon whose patterns of consistency, variability, contextual sensitivity, and breakdown revealed the organisation of underlying regulatory systems. Liddell’s longitudinal observations of sheep exemplified this approach (Liddell, 1947), as did Gantt’s multi-year case studies of individual dogs (Ramsden, 2018). By attending to the temporal dynamics, contextual modulation, and individual variability of behaviour, his group could infer inhibitory control, emotional reactivity, and social bonding. These processes could not be directly observed through physiological measurements alone. This methodological stance anticipated key visions in contemporary systems neuroscience, although contemporary neuroscience has not always acknowledged this historical continuity. The challenge of linking neural circuit activity to behaviour has become increasingly apparent as optogenetic and recording technologies enable unprecedented precision in manipulating and measuring neural dynamics (Krakauer et al., 2017; Jonas & Kording, 2017). Yet such precision is meaningful only if the behaviours under study are well-defined, theoretically grounded, and ecologically valid. Without careful characterisation of behavioural organisation—its structure, flexibility, and breakdown under stress—mechanistic inference remains indeterminate. A given pattern of neural activity can support multiple behaviours depending on context. Conversely, a given behaviour can be implemented through multiple neural configurations. This many-to-many mapping exposes the need to treat behaviour as an autonomous level of analysis with its own organisational principles (Gomez-Marin & Ghazanfar, 2019).

The experimental neurosis paradigm also revealed a second epistemological principle: pathology provides privileged access to regulatory organisation. This view, central to clinical medicine since the nineteenth century, was articulated systematically by Claude Bernard (1813–1878) and later by Walter Cannon (1871–1945) in the context of homeostasis (Bernard, 1865/1957; Cannon, 1932). By observing what fails first, under what conditions, and with what consequences, the observer can infer organisational features that remain invisible during normal function. In experimental neurosis research, behavioural breakdown under chronic conflict revealed the limits and failure modes of regulatory systems, including the conditions under which inhibitory control collapsed, the persistence of maladaptive patterns, and individual differences in vulnerability and resilience. These patterns could not have been inferred from studying learning under optimal conditions alone. They required the systematic disruption of normal regulation—what might be termed experimental psychopathology. This was a method for accessing organisational principles that remain invisible during normal function. This methodological commitment, however, brings significant ethical and practical challenges. The deliberate induction of suffering in animals for scientific purposes has long been contested, and standards for justifying such research have evolved considerably since the mid-twentieth century. Contemporary frameworks for animal research ethics require not only minimisation of suffering but also clear demonstration of translational relevance, ensuring that findings from animal models can meaningfully inform understanding or treatment of human conditions (Russell & Burch, 1959). Yet contemporary research continues to struggle with translating simplified conditioning models to human psychopathology (Heller, 2020). The history of experimental neurosis raises difficult questions in this regard. The relationship between animal behavioural disturbances and human psychiatric syndromes was never straightforward. Analogies drawn by researchers—between canine “neuroses” and human neurasthenia, between ovine hypervigilance and human anxiety—were often provisional and contested. These considerations raise the questions of how successfully experimental neurosis research achieved its translational aims and to what extent such aims should have constrained the methods employed.

These questions do not have simple answers, but the historical record suggests an important lesson: the value of animal models lies less in their ability to replicate specific human disorders than in their capacity to reveal general principles of adaptive and pathological regulation. Experimental neurosis research contributed to psychiatry not by producing animal “versions” of clinical syndromes. Instead, it revealed that chronic unresolvable conflict could induce persistent behavioural and physiological disturbances, that these disturbances reflected failures of regulatory integration rather than simple learning deficits, and that individual differences in temperament, developmental history, and social context modulated vulnerability to breakdown. These views—articulated through systematic observation of animal behaviour—shaped subsequent thinking about stress, anxiety, and resilience in ways that continue to inform clinical and translational research. Yet the paradigm also revealed a risk inherent in mechanistic reduction. The proliferation of simplified assays in mid-century stress research—e.g., forced swim tests, elevated plus mazes, social defeat protocols—was accompanied by a narrowing of theoretical focus. Behaviour was increasingly treated as an index of internal states (e.g., “anxiety-like behaviour,” “depressive-like behaviour”) rather than as a system whose organisation and breakdown were themselves the objects of study. This shift reflected broader changes in experimental culture, including the standardisation of laboratory animals, statistical methods prioritising group averages over individual trajectories, and the rise of psychopharmacology, which focused on drug effects measurable through simple behavioural endpoints (Koch, 2019; Ramsden, 2011). The result was a diminished capacity to capture the context-sensitivity, temporal dynamics, and individual variability that researchers like Liddell had exposed. Behaviour became, once again, subordinated to mechanism, no longer the primary interface through which regulation could be understood but merely an output to be correlated with manipulations. Contemporary reviews confirm that simple conditioning paradigms continue to fail in translating to human psychopathology, precisely because they cannot capture the emotional, cognitive, and contextual complexity of clinical disorders (Heller, 2020).

Contemporary efforts to revitalise translational psychiatry have recognised some of these limitations. The Research Domain Criteria (RDoC) framework, for instance, explicitly calls for multi-level analysis integrating behavioural, circuit, physiological, and molecular measures (Insel et al., 2010; Sanislow et al., 2010; Cuthbert & Insel, 2013). Similarly, proposals for “computational psychiatry” stress the need for formal models that link neural processes to behavioural dynamics through explicit mechanistic hypotheses (Maia & Frank, 2011). These frameworks acknowledge what the experimental neurosis tradition had already grasped: that explanation requires specifying how activity at one level generates organised phenomena at another, and that this cannot be achieved by reducing behaviour to mechanism without loss of explanatory content.

Yet these contemporary frameworks have not fully resolved the epistemological challenges revealed by the history of experimental neurosis. In particular, they have not consistently addressed the question of what counts as adequate behavioural characterisation. Measuring “social interaction time” or “entries into the open arm of an elevated plus maze” may provide quantitative endpoints, but do such measures capture the structured, context-sensitive, adaptively organised phenomena that constitute behaviour as a regulatory system? The experimental neurosis tradition suggests that they do not—that understanding behavioural regulation requires attention to temporal patterns, contextual modulation, developmental trajectories, and individual variability in ways that standardised assays often preclude.

This is not an argument against quantification or experimental control. It is an argument for recognising that behavioural phenomena are themselves structured, and that capturing this structure requires methods sensitive to organisation across multiple timescales and contexts. Liddell’s hybrid experimental–observational approach, combining controlled conditioning with naturalistic observation in social groups, exemplified one such method. Contemporary approaches such as automated behavioural tracking, dynamical systems modelling, and machine learning–based pattern recognition offer new technical possibilities for characterising behavioural organisation (Gomez-Marin & Ghazanfar, 2019). But these methods will fulfil their promise only if guided by theoretical commitments that treat behaviour not as an output, but as an autonomous, functionally organised domain that both constrains and is constrained by underlying mechanisms.

In sum, the epistemological lesson of early comparative psychopathology is twofold. First, behaviour is not reducible to mechanism without ontological loss: reducing it in this way eliminates the functional organisation that renders neurophysiological activity explanatorily relevant. Second, pathology reveals regulatory organisation in ways that normal function alone cannot, making the systematic study of behavioural breakdown essential for understanding adaptive regulation. These principles remain salient for contemporary neuroscience and psychiatry. They suggest that progress towards mechanistic explanation in mental disorder requires not only new technologies for the measurement and manipulation of neurophysiological activity but also renewed attention to behaviour as the irreducible interface through which regulation becomes scientifically accessible. The history of experimental neurosis exposes both the possibilities and the challenges of this endeavour. What remains is to articulate clearly what this history implies for contemporary neuroscience and psychiatry—not merely as a cautionary tale, but as a basis for conceptual reorientation.

## Conclusion: restoring behaviour to its ontological and epistemological status

This article has traced the emergence, elaboration, and subsequent transformation of experimental neurosis research from the early twentieth century through the mid-century period in which the paradigm fractured and was progressively marginalised. The reconstruction has been deliberately historical, but its implications are decisively contemporary. The trajectory that emerges is not one of cumulative progress, but of sustained conceptual contestation, methodological diversification, and eventual reductive narrowing. Yet the central thesis motivating this reconstruction extends beyond historical interest: behaviour possesses an irreducible ontological and epistemological status in the study of psychological regulation and its breakdown. The subordination of behaviour to neurophysiological mechanism—characteristic of much contemporary systems neuroscience—is therefore not a neutral methodological choice, but a conceptual stance that risks eliminating the very phenomena upon which scientific explanation depends.

The experimental neurosis paradigm took shape during a period in which behaviour was treated as a primary object of scientific inquiry, rather than as a proxy for more fundamental neural or physiological processes. Researchers such as Pavlov, Liddell, Gantt, and Masserman understood behaviour as the observable interface through which internal regulatory dynamics become accessible to systematic investigation. This stance did not reflect anthropomorphism or theoretical naïveté. It reflected a recognition, grounded in experimental practice, that regulatory processes can only be known through their behavioural manifestations. Neural activity, endocrine secretion, and autonomic responses acquire functional significance only by virtue of the behavioural contexts in which they occur and the organised patterns they sustain.

The conceptual foundations of comparative psychology were shaped by enduring tensions between mechanistic parsimony and behavioural richness, between reductionist explanation and systemic understanding. Early researchers confronted persistent challenges: individual variability, context-sensitivity, developmental change, and species-specific adaptation all resisted reduction to simple stimulus–response laws. These challenges did not disappear but were temporarily bracketed as experimental control and quantification became disciplinary imperatives. By the mid-century, the result was a methodological landscape in which behaviour was increasingly treated as an index rather than a system—a dependent variable to be correlated with physiological measures, rather than an autonomous domain exhibiting its own organisational principles.

The experimental neurosis paradigm, particularly in its earlier and more exploratory phases, exemplified a different approach. Pavlov’s demonstration that behavioural regulation could break down under conditions of chronic conflict revealed that behaviour is not merely learned association, but a dynamically regulated process vulnerable to systemic failure. Liddell’s expansion of this framework showed that behavioural dysregulation could be studied systematically without being reduced to simplified metrics. Masserman’s integration of Pavlovian methods with psychoanalytic concepts further showed how experimental procedures could be designed to reveal motivational conflict and its resolution. Taken together, these approaches shared a common commitment. That is, behaviour, observed across multiple contexts and timescales, provides access to regulatory organisation that physiology alone cannot disclose.

Experimental neurosis eventually gave way to more overtly reductionist frameworks. The proliferation of standardised behavioural assays in the mid-to-late twentieth century reflected a broader shift in epistemic values. From exploratory observation to hypothesis testing; from longitudinal case studies to cross-sectional group comparisons; and from behavioural complexity to simplified endpoints. This transformation was not merely technical, but conceptual. It mirrored changing assumptions about what behaviour is and what it can reveal. Behaviour came to be understood primarily as an output of underlying mechanisms, rather than as the organisational level at which such mechanisms acquire functional significance. The consequences of this shift persist in contemporary translational neuroscience, where the gap between increasingly precise neural manipulation and limited behavioural understanding remains a major obstacle to mechanistic explanation (Krakauer et al., 2017; Jonas & Kording, 2017; Gomez-Marin & Ghazanfar, 2019).

The trajectory reconstructed here suggests that this obstacle is not merely technical—a matter of developing better measurement tools or more sophisticated statistical methods—but fundamentally conceptual (Heller, 2020). It reflects a failure to recognise that behaviour occupies an irreducible position in scientific explanation.

First, behaviour is ontologically primary. Not as a more fundamental level of being than the neural or the molecular, but in the sense established in the preceding section. It is the level at which lower-level activity acquires its functional significance, and thus what renders mechanism intelligible as organised functional activity rather than what merely comes after it. Neural circuits do not produce behaviour in the manner of a machine generating output. Rather, neural activity forms part of a system spanning brain, body, and environment, the organisation of which is manifest in behavioural patterns. Understanding this organisation therefore requires attending to behaviour not as a derivative phenomenon, but as the level at which regulatory processes achieve functional integration.

Second, behaviour is epistemologically indispensable. Its observability is not a limitation, but an epistemic advantage. Behaviour provides the criteria by which we judge whether neural activity is adaptive or pathological, whether it constitutes learning or failure to learn, and whether it reflects appropriate or inappropriate responsiveness to context. Without such criteria, mechanistic explanation becomes indeterminate: neural activity can be described in exquisite detail, yet its functional significance—what it is *for*—remains opaque.

Third, behavioural complexity is scientifically tractable. The objection that behaviour is “too complex” to serve as the basis for mechanistic explanation misconstrues the nature of explanation itself. Explanation does not require reducing phenomena to their simplest constituents; it requires showing how organised activity at one level gives rise to structured patterns at another. Early comparative psychopathology showed that behavioural organisation can be systematically characterised and experimentally accessed. Contemporary methods extend rather than replace this tradition, providing new technical means for capturing behavioural structure across timescales and contexts.

Fourth, pathology reveals organisation. Experimental neurosis exemplified a methodological principle that remains essential: regulatory systems are often most clearly understood through their failure modes. Observing what breaks down, under what conditions, and with what consequences affords access to organisational features that remain invisible during normal function. This principle applies as much to contemporary neuroscience as it did to mid-century stress research. Circuit-level manipulations that induce behavioural deficits are informative precisely because they reveal how neural activity contributes to organised behavioural regulation. Interpreting such deficits, however, requires a sophisticated understanding of behavioural organisation itself—what counts as disruption, how it manifests across contexts, and how it differs from normal variability.

These considerations converge on a single claim: restoring behaviour to its proper ontological and epistemological status is a necessary condition for progress in systems neuroscience and translational psychiatry. This is not a call to abandon mechanistic investigation, nor to return to pre-molecular methods. It is a call to recognise that mechanistic explanation is explanatory only insofar as it specifies how activity at one level generates organised phenomena at another—and that behaviour, as the level at which neural and physiological processes acquire functional meaning, cannot be bypassed or subordinated without loss of explanatory power.

What would such a restoration look like in practice? The history of experimental neurosis offers several concrete directions. Their content overlaps the agenda of contemporary computational ethology and naturalistic neuroscience, but the historical case supplies something those programmes cannot provide on their own: evidence that these directions are not merely aspirational. Each was realised, and to a considerable degree integrated, in the early-to-mid-twentieth-century tradition before being displaced. The lesson is therefore not that an integrative behavioural science is infeasible, but that its earlier eclipse was driven by disciplinary incentives—standardisation, throughput, and the prestige of mechanism—rather than by any established inadequacy of the approach itself.

First, integrate laboratory and naturalistic observation. Liddell’s mimic farm showed that experimental control and ecological validity need not be mutually exclusive. Contemporary research would benefit from hybrid designs that combine precise experimental manipulation with systematic observation of behaviour across social, developmental, and environmental contexts. This integration is particularly crucial in the study of psychopathology, where symptoms manifest differently depending on social structure, environmental demands, and temporal dynamics.

Second, attend to temporal organisation. Behavioural regulation unfolds across multiple timescales: milliseconds of motor control, seconds of decision-making, hours of state transitions, days of learning, and months or years of developmental change. Early comparative psychopathology—particularly the longitudinal approaches of Gantt (Ramsden, 2018) and Liddell—recognised that understanding regulation requires tracking behaviour across these timescales. Contemporary research, by contrast, too often relies on brief snapshots, missing the temporal dynamics through which adaptive and maladaptive patterns emerge, stabilise, and transform.

Third, take individual variability seriously. The standardisation imperative in modern animal research—genetic uniformity, controlled environments, and averaged outcomes—aims to reduce “noise” and increase reproducibility (Ramsden, 2011). Yet, as early researchers recognised, individual differences are not noise but signal. They reveal the range of regulatory possibilities, the factors that confer vulnerability or resilience, and the developmental trajectories through which stable patterns of behaviour emerge. Understanding psychopathology therefore requires attention not only to group means, but to individual trajectories of adaptation and breakdown.

Fourth, develop theory-driven behavioural characterisation. Much contemporary research relies on behavioural assays adopted for pragmatic reasons—ease of measurement, historical precedent, or commercial availability—without a clear theoretical account of what those assays are intended to capture. The experimental neurosis tradition suggests an alternative: designing behavioural paradigms explicitly to reveal organisational features of interest (such as inhibitory control, motivational conflict, or social regulation), and validating them through convergent evidence across measures, contexts, and timescales.

Fifth, sustain bidirectional translation. The relationship between experimental models and clinical phenomena was never unidirectional. Animal research informed psychiatric theory, but clinical observation and conceptual frameworks equally shaped experimental design and interpretation. Contemporary translational research would benefit from restoring this reciprocity: allowing clinical views to guide model construction and evaluating experimental findings in terms of their capacity to illuminate clinical phenomena, rather than merely to correlate with diagnostic categories.

The practical directions outlined here rest on a single conceptual foundation: behaviour is an autonomous domain with its own organisational principles, not a derivative output of underlying mechanisms. Recognising this does not diminish the importance of neural, molecular, or genetic levels of analysis. Rather, it clarifies their explanatory role. These levels are components of larger regulatory systems whose functional organisation is manifest in behavioural patterns and accessible through systematic behavioural investigation. Mechanistic explanation is therefore not achieved by bypassing behaviour, but by showing how organised activity at lower levels generates and sustains behavioural regulation across contexts and timescales.

The historical case of experimental neurosis illustrates both the promise and the difficulty of this stance. Early comparative psychopathologists showed that behavioural pathology could be systematically produced, characterised, and studied across species and contexts. They developed methods for integrating behavioural observation with physiological measurement, tracking individual trajectories over extended periods, and revealing regulatory organisation through its breakdown. At the same time, the paradigm exposed persistent challenges: defining pathology, negotiating cross-species analogy, balancing experimental control with ecological validity, and confronting the ethical limits of inducing suffering. These tensions were never fully resolved, and their unresolved status contributed to the paradigm’s eventual marginalisation. Yet their persistence does not undermine the approach; it clarifies what is at stake when behaviour is taken seriously as a scientific object.

Contemporary neuroscience confronts analogous challenges in altered form. The question is no longer whether animal behaviour can inform psychopathology, but how behavioural phenomena can be linked to neural mechanisms in ways that are both explanatorily robust and translationally meaningful—a problem that demands conceptual reorientation as much as technological innovation. The history reconstructed here does not resolve it, but it clarifies the choice: either behaviour is treated as a legitimate object of inquiry in its own right, or it is subordinated to supposedly more fundamental levels of analysis at the cost of explanatory coherence. Early comparative psychopathologists grasped what contemporary neuroscience risks overlooking—that organisms are not mechanisms but organised systems whose functional properties emerge from, yet are not reducible to, their components, and that behaviour is the level at which this organisation becomes visible and scientifically interrogable.

The conclusion, then, is both historical and programmatic. Historically, the experimental neurosis paradigm showed that dysregulation can be systematically studied through careful attention to behavioural organisation and its breakdown. Programmatically, it suggests that progress in understanding psychopathology requires restoring behaviour to centrality: not as an outcome to be explained away, but as the interface through which explanation proceeds; not as a symptom of underlying pathology, but as the domain in which regulatory organisation and its failure become scientifically accessible. Whether contemporary neuroscience will fully embrace this reorientation remains an open question. The history traced here suggests, however, that without it, the explanatory ambitions of mechanistic psychiatry will remain unfulfilled. Behaviour is not what neuroscience leaves behind on the way to mechanism; it is what makes mechanism intelligible at all.

## Data Availability

No data was used for the research described in the article.
